# Activin A Modulates Betaglycan Shedding via the ALK4-SMAD3-Dependent Pathway in Endometriotic Cells

**DOI:** 10.3390/biom12121749

**Published:** 2022-11-24

**Authors:** Agnes N. Mwaura, Muhammad A. Riaz, Jane B. Maoga, Ezekiel Mecha, Charles O. A. Omwandho, Georgios Scheiner-Bobis, Ivo Meinhold-Heerlein, Lutz Konrad

**Affiliations:** 1 Faculty of Medicine, Center of Gynecology and Obstetrics, Justus-Liebig-University, D-35392 Giessen, Germany; 2 Department of Biochemistry, University of Nairobi, Nairobi P.O. Box 30197-00100, Kenya; 3 Department of Health Sciences, Kirinyaga University, Kerugoya P.O. Box 143-10300, Kenya; 4 Institute for Veterinary Physiology and Biochemistry, School of Veterinary Medicine, Justus-Liebig-University, D-35392 Giessen, Germany

**Keywords:** activin A, betaglycan, SMAD2/3, MMPs, endometriosis

## Abstract

The TGF-β superfamily members, activins and inhibins, are mainly involved in cell proliferation, cell survival, invasion, immune surveillance, and lesion growth in endometriosis. Herein, we investigated the modulation of the TGF-β type III receptor (betaglycan or BG) by activin A and inhibin A in endometriosis in vitro. Often, BG undergoes ectodomain shedding releasing soluble BG (sBG) which frequently antagonizes TGF-β signaling. The effects of activin A on BG shedding and signaling pathways involved were evaluated with the inhibitors LY364947 and SIS3, siRNA knockdown in human endometrial cells (12Z, THESC, Ishikawa, and primary stromal cells) and were quantified with BG ELISAs. The effects of activin A and inhibin A on the secretion of MMP2 and MMP3 were analyzed using ELISAs. The effects of activin A on the BG expression were analyzed using RT-qPCR and western blot. The CCK-8 and BrdU assays were used to evaluate the effects of the recombinant BG on cell viability and proliferation. Activin A stimulation resulted in a significant time- and dose-dependent reduction in BG shedding, which was found to be activin A/ALK-4/SMAD3- but not SMAD2-dependent. Activin A increased the BG mRNA expression but had no effect on the protein expression. Likewise, inhibin A was found to block BG shedding. Activin A, but not inhibin A, significantly enhanced the secretion of MMP2 and MMP3. The recombinant BG had no effect on the viability and proliferation of endometriotic cells. Together, these observations support a novel role for activin A with BG in modulating the TGF-β superfamily ligands in endometrial cells in vitro.

## 1. Introduction

Endometriosis is associated with the occurrence of endometrial tissue outside the uterine cavity, primarily in the ovaries, pelvic peritoneum, bladder, bowel, and retro-vaginal septum [1]. It affects about 0.7–8.6% of reproductive age women and is frequently associated with chronic pelvic pain, dyspareunia, dysmenorrhea, dyschezia, and infertility [1,2,3,4]. Endometriosis involving the ovary is the most common form (17–44%) and affects up to 50% of women suffering from infertility and abdominal pain [5]. Several theories have been proposed to explain the development and survival of ectopic endometrial lesions, however, the retrograde menstruation theory is the most widely accepted theory [6] and postulates that endometriosis arises from the passage of endometrial fragments via the fallopian tubes during menstruation and consequent implantation at ectopic sites.

The transforming growth factor beta (TGF-β) superfamily ligands comprise structurally related proteins including the TGF-βs, activins, and inhibins [7]. These molecules are essential for normal cellular functions as well as the pathophysiology of numerous diseases including endometriosis [8,9,10,11]. These cytokines and several other biologically active agents modulate the proteolytic activity of matrix metalloproteinases (MMPs) including MMP2, 3, and 9 [12]. Particularly, MMPs are involved in cyclic changes of the endometrial structure and thickness during menstruation and dysregulation of MMP2, and MMP3 has previously been implicated in the pathogenesis of endometriosis [12].

Activin and inhibin share comparable β subunits, with activins occurring as ββ homodimers, whereas inhibins are αβ heterodimers [13]. They are key players in physiological processes like germ cell development, oocyte maturation, follicular development, ovulation, decidualization, endometrial receptivity and embryo implantation, placentation, as well as endometrial repair after menstruation [13,14,15]. The aberrant expression of activins and inhibins and their signaling pathways has already been reported in women with endometriosis [10,16,17,18]. Consequently, an apt regulation of these pathways is mandatory at all levels [10].

TGF-β superfamily co-receptors are vital as they determine whether a certain cell responds to a ligand or whether they can function as a decoy and thus antagonize signaling [19]. Betaglycan (BG) is a ubiquitously expressed transmembrane co-receptor for some TGF-β superfamily ligands including TGF-βs and inhibins [20,21] and establishes the potency of its ligands on their target cells [22]. BG possess additional ligand-dependent and -independent roles such as the regulation of reproduction and tumor suppression [23,24,25,26]. The membrane-bound BG (mBG) undergoes proteolytic ectodomain cleavage, a process termed shedding, releasing a soluble domain (sBG) that can be detected in extracellular matrix and body fluids [26,27,28,29,30].

Recently, we demonstrated that BG modulates TGF-β signaling in rat Sertoli cells [31] and in human endometrial cells [30]. Apart from TGF-βs, BG also binds inhibin with high affinity and is a key determinant of inhibin potency especially on pituitary LβT2 gonadotrope cells [32]. Inhibin antagonizes activin signaling via the association of its β subunit with a single type II activin receptor (ACVR2) and through the association of its α subunit with BG to form an inactive inhibin-receptor complex. This complex is incapable of signal transduction as it leads to the sequestration of ACVR2, thus inhibiting activin signaling [15,33]. Accordingly, we investigated the involvement of activin A and inhibin A in BG shedding and expression, the molecular mechanisms involved, and whether or not BG affects proliferation of endometriotic epithelial cells. We further explored the influence of activin A and Inhibin A on the secretion of MMP2 and MMP3 in endometriotic epithelial cells.

## 2. Materials and Methods

### 2.1. Cells

The immortalized and well-characterized 12Z cell line [34,35] was kindly provided by Prof. Anna Starzinski-Powitz (Department of Biology, University of Frankfurt, Frankfurt, Germany). Immortalized stromal THESC cells [36] were purchased from ATCC (Cat. No. CRL-4003) and the endometrial epithelial Ishikawa cell line was purchased from Sigma Aldrich, St. Louis, MO, USA (Cat. No. 99040201).

Primary human endometrial stromal cells (PHESCs) were obtained from the eutopic endometrial tissue of a 45-year-old woman who underwent surgery by an abdominal total hysterectomy due to endometriosis and uterine myoma. The patient had not received hormonal therapy before surgery. A preoperative informed consent form was obtained from the patient. The study was approved by the Ethics Committee of the Medical Faculty of the Justus Liebig University, Giessen, Germany (registry number 95/09), and the experiments were performed in accordance with relevant guidelines and regulations. PHESCs were isolated and cultured as previously described [37,38] with some modifications. Briefly, endometrial tissue was minced into small pieces (1–3 mm) and digested in a DMEM/F12 medium containing 1 mg/mL of *Clostridium histolyticum* collagenase at 37 °C for 2 h in a shaking water bath, and the suspension passed through 70 μm cell strainers. The filtrate containing both endometrial stromal and epithelial cells was centrifuged at 500× *g* for 5 min and the cell pellet was re-suspended in a DMEM/F12 medium supplemented with a 10% FCS, 1% pen-strep and 1% insulin, transferrin, and selenium (ITS) solution. A differential method was used to separate PHESCs from epithelial cells since stromal cells, unlike epithelial cells, adhere firmly to cell culture plates after 18 h [39,40]. After pre-plating for 18 h, the supernatant containing floating epithelial cells and other types of cells was removed, and the remaining stromal cells were maintained in a humidified incubator at 37 °C and 5% CO_2_. Purity of PHESCs was assessed by a morphological examination with CD10 and CD248 (both stain positive for stromal cells), and mucin 1 (positive for epithelial but negative for stromal cells). The purity of PHESCs was greater than 99.5%. Cultured primary stromal cells at passages 4–5 were used for further analysis.

### 2.2. Cell Culture

Cells were maintained in the medium as follows: 12Z cells in DMEM 4.5 g/L glucose, supplemented with 10% FCS, 2 mM glutamine, and 1% pen-strep; THESC and primary human endometrial stromal cells in DMEM/F12 with 2 mM glutamine, supplemented with 10% FCS, 1% pen-strep, and 1% ITS solution; Ishikawa cells in MEM, supplemented with 5% FCS, 1% non-essential amino acids, 2 mM glutamine, and 1% pen-strep. All cell types were cultured in a humidified incubator at 37 °C and 5% CO_2_, and the medium was routinely renewed every 3–4 days. Cells were washed once with 1× PBS without Ca^2+^ and Mg^2+^ before detachment with 0.25% accutase and were passaged at about 80% confluence. All cell culture reagents were purchased from Invitrogen/ThermoFisher Scientific (Karlsruhe, Germany).

### 2.3. Recombinant Proteins, Inhibitors, and ELISAs

The following materials were used: recombinant human activin A and follistatin (both from Promokine, Heidelberg, Germany); inhibin A (Novus Biologicals, Centennial, USA); recombinant human betaglycan (R&D Systems, Wiesbaden, Germany); anti-TGF-β RIII antibody (Cell signaling technology, Frankfurt am Main, Germany); LY364947 (Sigma Aldrich, St. Louis, MO, USA); SIS3 (Merck, Darmstadt, Germany); human TGF-beta RIII DuoSet ELISA kit (DY242, range 156–10,000 pg/mL); Human MMP2 DuoSet ELISA kit (DY902, range 0.6–20 ng/mL); Human Total MMP3 DuoSet ELISA kit (DY513, range 31.3–2000 pg/mL). All ELISA kits were purchased from R&D Systems.

### 2.4. Treatment of Cells with Various Agents

Cells (2 × 10^5^ cells) were cultured in 6-well plates and serum-starved (1% FCS) for 24 h and treated in duplicates with activin A (5–50 ng/mL), follistatin (200 ng/mL), inhibin A (5–50 ng/mL), or recombinant BG (10–200 ng/mL). For inhibition studies, cells were pre-treated with the respective inhibitors for 2 h (10 µM LY364947 or 5 µM SIS3 diluted in 0.01% DMSO) prior to stimulation with activin A (25 ng/mL). Negative controls consisted of untreated samples without the recombinant proteins/inhibitor or containing only the vehicle (0.01% DMSO).

### 2.5. Betaglycan, MMP2 and MMP3 ELISAs

The collection of supernatants and ELISAs was performed as previously described [30].

### 2.6. Western Blot

Endometriotic 12z cells (2 × 10^5^ cells) were cultured in 6-well plates and serum-starved (1% FCS) for 24 h and treated in duplicates with activin A (25 and 50 ng/mL) for 24 h. Cells were harvested and lysed in a 500 μL lysis buffer (1 mM EDTA, 0.5% Triton X-100, 5 mM NaF, 6 M urea, 10 μg/mL Leupeptin, 10 μg/mL Pepstatin, 100 μM PMSF, 3.0 μg/mL Aprotinin, 2.5 mM Sodium Pyrophosphate, 1 mM activated Sodium Orthovanadate in PBS, pH 7.2–7.4, R&D Systems) for 5 min and lysates were collected with a cell scraper. Protein quantification was performed with the Bicinchoninic acid (BCA) protein assay (Pierce, Thermo Scientific). A total of 15 μg protein of the protein lysates were loaded onto an SDS-PAGE and blotted onto Immobilon-P PVDF membranes (Merck-Millipore, Darmstadt, Germany) at 0.5 V/cm2 for 30 min. The PVDF membranes were then blocked for 1 h with 5% BSA in a Tris–buffered saline 0.5% Tween (TBS-T) solution at room temperature and probed with an anti-TGF-β RIII antibody (110 KDa; 2519, cell signaling) overnight at 4 °C. The membranes were then washed three times with TBS-T for 10 min and then incubated with an anti-rabbit HRP-conjugated secondary antibody (7074, Cell Signaling, Frankfurt am Main, Germany) for 1 h at room temperature. Following four times washing with TBS-T for 5 min, a chemiluminescence signal was generated with the SignalFire ECL Kit (Cell signaling) and captured on X-ray film. To ensure equal protein loading per lysate sample, the membranes were stripped and probed again for vinculin (116 kDa; V9264, Sigma).

### 2.7. SiRNA Transfection, RNA Isolation and Real Time-qPCR

Endometriotic 12Z cells (2 × 10^5^ cells) were cultured in 6-well plates and serum-starved (1% FCS) for 24 h and treated in duplicates with activin A (25 and 50 ng/mL) for 2 h. RNA was isolated and real time qPCR was carried out. SiRNA transfection, RNA isolation and RT-qPCR procedures were performed as previously described [30].

### 2.8. CCK-8 Cell Viability and BrdU Proliferation Assays

Cell viability assay was performed using the cell counting kit-8 (CCK-8) following the manufacturer’s instructions. Briefly, approximately 4000 endometriotic 12Z cells were seeded in a 96-well cell culture plate and incubated in a humidified atmosphere at 37 °C and 5% CO_2_. After 24 h, the complete medium was replaced with a starvation medium (1% FCS) for another 12 h. The culture medium was replaced by serial concentrations of human recombinant BG (10–200 ng/mL) and diluted in the starvation medium. Cells were cultured for 21 h and 10 μL of the CCK-8 reagent was added into each well and incubated for an additional 3 h. Cell viability was determined by taking optical density (OD) values using an infinite^®^ 200 microplate reader set at a wavelength of 450 nm. Cell viability was presented as a percentage of each concentration relative to control. The colorimetric BrdU assay (Abcam, Cambridge, UK) was used to analyze the proliferation of endometriotic cells following treatment with human recombinant BG (10–200 ng/mL) as previously described [31].

### 2.9. Statistical Analyses

Each experiment was performed at least three times in duplicates. Results were presented as means ± SEMs (SEM = standard error of the mean). Statistical analyses were performed with the GraphPad Prism software (Version 8.0, GraphPad Inc., La Jolla, CA, USA). Statistical comparisons of the means among multiple groups were performed by one-way analysis of variance (ANOVA) followed by Dunnett’s post hoc tests. Differences were considered statistically significant at *p* ≤ 0.05.

## 3. Results

### 3.1. Modulation of BG Shedding and Expression by Activin A in Endometrial Cells

**Figure 1:**
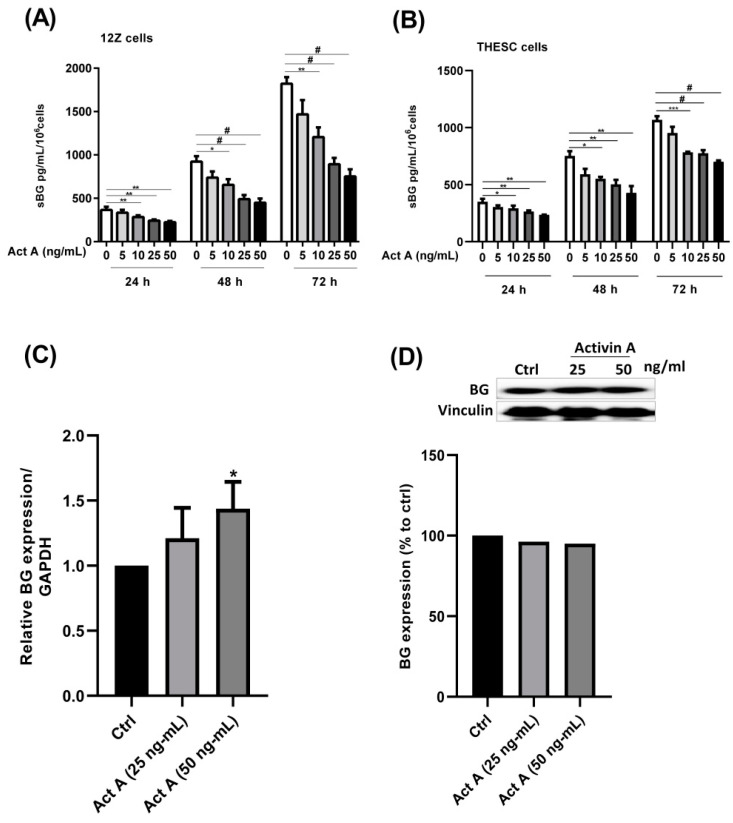
Time- and concentration-dependent effects of activin A on BG shedding and expression in epithelial and stromal cells. Epithelial 12Z (**A**) and stromal THESC cells (**B**) were stimulated with increasing concentrations of activin A (5–50 ng/mL) for 24, 48, and 72 h and supernatants were analyzed by sBG ELISAs. The 12Z cells were also treated with 25 and 50 ng/mL of activin A and BG mRNA (**C**) and protein expression (**D**) and were analyzed by RT-qPCR and western blot after 2 h and 24 h, respectively. Activin A treatment significantly suppressed BG shedding in a time- and concentration-dependent manner in both epithelial and stromal cells. Moreover, the activin A stimulation significantly increased BG mRNA but had no effect on the BG protein expression. Untreated cells were used as controls. Each bar represents the mean ± SEM of 3 independent experiments performed in duplicate. Dunnett’s test was used for statistical analysis; * *p* ≤ 0.05; ** *p* < 0.01; *** *p* < 0.001; ^#^ *p* < 0.0001; sBG, soluble betaglycan; Act A, activin A.

**Figure 2:**
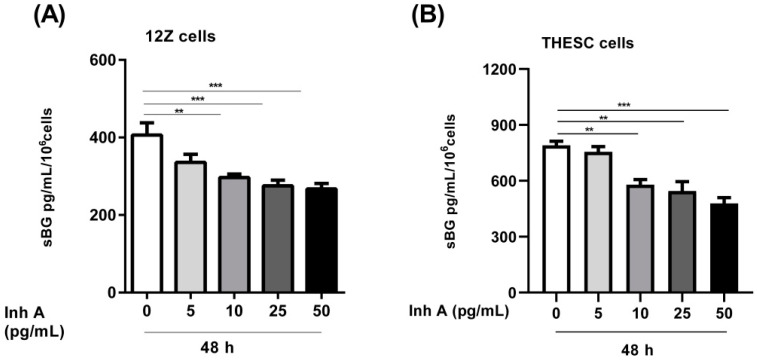
Concentration-dependent effects of inhibin A on BG shedding in epithelial and stromal cells. Epithelial 12Z (**A**) and stromal THESC cells (**B**) were stimulated with increasing concentrations of inhibin A (5–50 ng/mL) for 48 h and supernatants were analyzed by sBG ELISAs. Inhibin A treatment significantly reduced BG shedding in 12Z and THESC cells in a concentration-dependent manner. Untreated cells were used as controls. Each bar represents the mean ± SEM of 3 independent experiments performed in duplicate. Dunnett’s test was used for statistical analysis; ** *p* < 0.01; *** *p* < 0.001; sBG, soluble betaglycan; Inh A, inhibin A.

In the present study we investigated whether activin A and inhibin A regulate BG shedding in endometrial cells. There was a significant time- and dose-dependent decrease in BG shedding following the stimulation of epithelial 12Z cells with varying concentrations of activin A (Figure 1A). An approximately 10–60% decrease was observed with 5–50 ng/mL of activin A after 24, 48, and 72 h (Figure 1A). Likewise, a 10–45% decrease was observed with 5–50 ng/mL of activin A after 24, 48, and 72 h in stromal THESC cells (Figure 1B). Generally, a stronger reduction of BG shedding was observed in activin A-treated epithelial 12Z cells compared with the stromal THESC cells (Figure 1A,B). Additionally, the BG mRNA expression increased 1.4-fold following a treatment with 50 ng/mL of activin A (Figure 1C). Conversely, no significant effects were observed on the membrane-bound BG protein expression (Figure 1D). Furthermore, the treatment of 12Z and THESC cells with inhibin A resulted in a substantial reduction in BG shedding (Figure 2A,B). Collectively, these results demonstrate that activin A and inhibin A treatment significantly reduce BG shedding in a time- and concentration-dependent manner in human epithelial and stromal cells.

### 3.2. Modulation of BG Shedding by Activin A and Inhibin A in Different Cell Types

**Figure 3:**
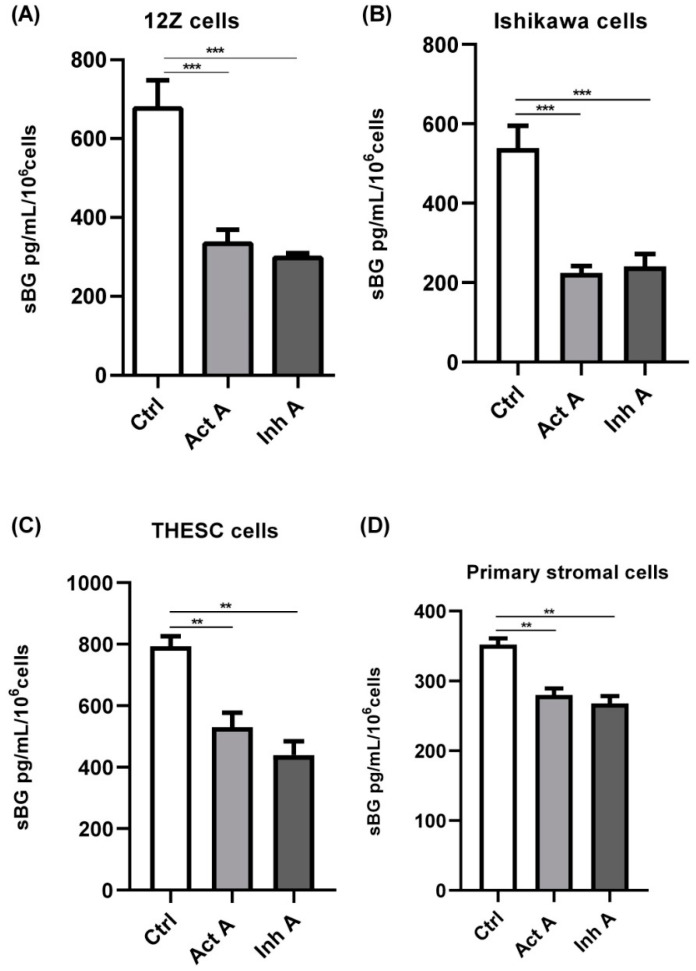
Activin A and inhibin A regulate BG shedding in different cell types. Endometriotic epithelial 12Z (**A**), endometrial epithelial Ishikawa (**B**), primary human endometrial stromal (**C**), and endometrial stromal THESC (**D**) cells were treated with activin A or inhibin A (25 ng/mL) for 48 h and supernatants were analyzed by sBG ELISAs. Both activin A and inhibin A significantly attenuated BG shedding in all four cell types. Untreated cells were used as controls (ctrl). Each bar represents the mean ± SEM of 3 independent experiments performed in duplicate. Dunnett’s test was used for statistical analysis; ** *p* < 0.01; *** *p* < 0.001; sBG, soluble betaglycan; Ctrl, control; Act A, activin A; Inh A, inhibin A.

Besides the epithelial endometriotic 12Z and stromal THESC cells, we also examined BG shedding in epithelial Ishikawa cells as well as primary human endometrial stromal cells. Both activin A and inhibin A significantly reduced BG shedding in epithelial 12Z cells (~50% and 55%, respectively; Figure 3A), Ishikawa cells (~60% and 55%, respectively; Figure 3B), primary stromal cells (~20% and 25%, respectively; Figure 3C), as well as stromal THESC cells (~35% and 45%, respectively; Figure 3D).

In summary, the epithelial cells exhibited a stronger reduction in BG shedding compared to stromal cells, 55% vs. 31%, respectively (Figure 3A–D). Moreover, the untreated stromal THESC cells released more sBG compared to the untreated epithelial Ishikawa cells (1.5-fold) and primary stromal cells (2.3-fold). Overall, these results confirm that activin A and inhibin A significantly suppress BG shedding in both endometrial epithelial and stromal cells.

### 3.3. Involvement of Activin Receptor Type-1B (ALK-4) in BG Shedding

**Figure 4:**
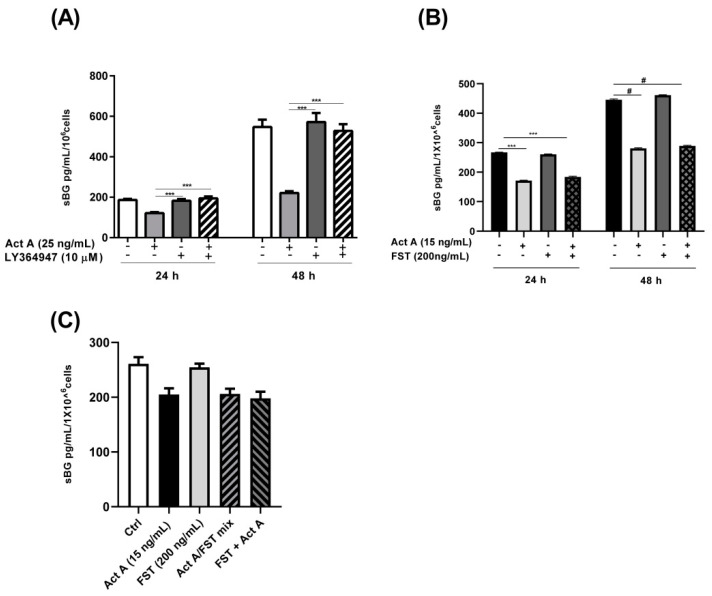
Activin A-mediated reduction in BG shedding is TGF-β type 1 receptor (ALK-4)-dependent but follistatin-independent. Epithelial 12Z cells were pre-incubated in the absence or presence of the ALK-4/5 inhibitor, LY364947 (10 µM) (**A**), or follistatin (200 ng/mL) for 2 h **(B**) or 30 min (**C**) prior to stimulation with activin A (25 ng/mL or 15 ng/mL) for 24 h and 48 h, or Activin A-follistatin mixture (**C**) for 24 h and supernatants were analyzed by sBG ELISAs. LY364947 completely abolished the reduction in BG shedding induced by activin A, whereas follistatin had no effect. Untreated cells or cells treated with DMSO (0.01%) were used as controls. Each bar represents the mean ± SEM of 3 independent experiments performed in duplicate. Dunnett’s test was used for statistical analysis; *** *p* < 0.001; # < 0.0001; sBG, soluble betaglycan; Act A, activin A; FST, follistatin; mix, mixture.

Next, we explored the signaling mechanisms involved in activin A-mediated reduction in BG shedding in endometriotic epithelial cells. Since signaling of the dimeric activin occurs through type I and type II serine-threonine kinase receptors [41], we investigated the involvement of the activin receptor type-1B (ACVRB1 or ALK-4) in BG shedding. Endometriotic 12Z cells were pre-treated with LY364947, which selectively inhibits ALK-4/5, prior to stimulation with activin A. Pre-treatment with LY364947 completely abrogated the activin A-induced reduction in BG shedding after 24 and 48 h (Figure 4A), thus, phosphorylation and activation of ACVRB1 is required for an activin A-mediated decrease in BG shedding. Besides the ALK-4 inhibitor, we also evaluated the effects of follistatin, an activin-binding and -neutralizing protein, on the activin A-mediated reduction in BG shedding. Contrary to the ALK-4 inhibitor, pre-treatment of 12Z cells with follistatin prior to the activin A treatment had no effect on the activin A-induced regulation of BG shedding (Figure 4B). Moreover, simultaneous addition of activin-follistatin mixture did not have any effects on BG shedding (Figure 4C).

### 3.4. Involvement of SMAD2/3 in Activin A-Mediated Reduction in BG Shedding

**Figure 5:**
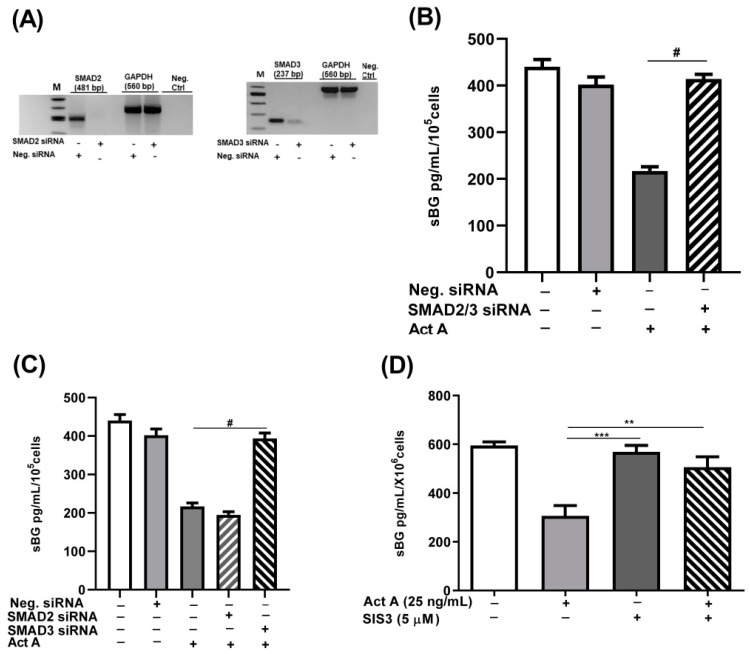
Activin A-mediated reduction in BG shedding is SMAD3- but not SMAD2-dependent. Epithelial 12Z cells were plated in 6-well plates and transfected with SMAD2 siRNA, SMAD3 siRNA, or negative control siRNA. RT-qPCR of the gene silencing efficiency for each siRNA was performed to ascertain the silencing of SMAD2 and SMAD3 (**A**). SMAD2/3 double-silenced and single-silenced 12Z cells were subsequently stimulated with activin A (25 ng/mL) for 48 h and supernatants were analyzed by sBG ELISAs. SMAD2/SMAD3 double gene knockdown significantly abrogated the activin A-dependent reduction in BG shedding (**B**). Single gene knockdown revealed that only the silencing of SMAD3 and not of the SMAD2 gene blocked the activin A-mediated effects (**C**). These results were further corroborated using the specific inhibitor of SMAD3, SIS3 (5 μM) (**D**). Untreated cells and cells treated with Neg. siRNA or DMSO (0.01%) were used as controls. Each bar represents the mean ± SEM of 3 independent experiments performed in duplicate. Dunnett’s test was used for statistical analysis; ** *p* < 0.01; *** *p* < 0.001; ^#^ *p* < 0.0001; siRNA, small interfering RNA; Neg. siRNA, negative control siRNA; sBG, soluble betaglycan; Act A, activin A.

The recruitment and activation of type II and type I activin receptors by activin A results in the phosphorylation and activation of SMAD2/3 in a canonical pathway [41]. Accordingly, we explored the involvement of the canonical SMAD signaling pathway by silencing SMAD2 and SMAD3 genes using siRNAs. The SMAD2/SMAD3 double-silenced epithelial 12Z cells were treated with activin A and sBG were quantified by ELISAs. We demonstrated that the SMAD2/SMAD3 double gene knockdown significantly and completely counteracted the activin A-dependent reduction in BG shedding (Figure 5B).

Subsequently, we investigated the individual contribution of each SMAD using SMAD2 and SMAD3 siRNA silencing and a SMAD3 inhibitor, SIS3, followed by activin A treatment. Analysis of individual knockdowns revealed that the silencing of SMAD3 but not SMAD2 abrogated activin A-mediated reduction in BG shedding by 98% (Figure 5C). These results were further validated by the SIS3 inhibitor which showed that the SMAD3 inhibition abolished ~85% of the effects of activin A in reducing BG shedding (Figure 5D). Concisely, these results imply that the canonical TGF-β pathway involving activin A/ACVRB1/SMAD3 signaling is required in activin A-mediated reduction in BG shedding.

### 3.5. Influence of Activin A and Inhibin A in MMP2 and MMP3 Secretion

Activin A and inhibin A are involved in the regulation of embryo implantation and trophoblast invasion mainly via their effects on MMPs, which are principal mediators of decidualization [42]. Moreover, the increased expression and activity of MMPs such as MMP2, MMP3, and MMP9 has been reported in endometriosis [12]. Thus, we investigated the effects of activin A and inhibin A on the secretion of MMP2 and MMP3 in endometriotic 12Z cells.

**Figure 6:**
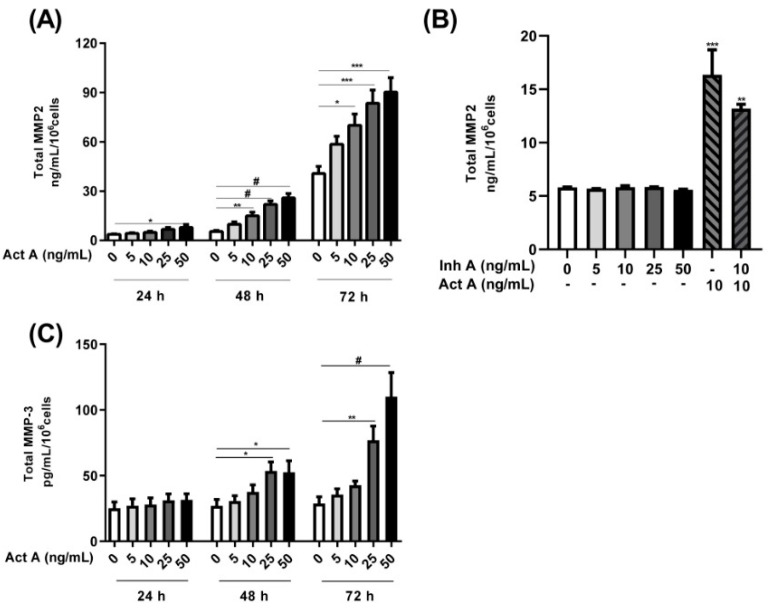
Time and concentration-dependent effects of activin A and inhibin A treatment on MMP2/3 secretion. Endometriotic 12Z cells were stimulated with increasing concentrations of activin A (5–50 ng/mL) for 24, 48, and 72 h and supernatants were analyzed by MMP2 (**A**,**B**) and MMP3 (**C**) ELISAs. Cells were also treated with inhibin A, with (10 ng/mL) or without activin A for 48 h and supernatants were analyzed by MMP2 ELISAs (**B**). Activin A treatment significantly augmented MMP2 and MMP3 secretion in a time- and concentration-dependent manner (**A**,**C**). Inhibin A treatment of the cells had no effect on MMP2 secretion although the simultaneous addition of inhibin A and activin A resulted in a moderate reduction in the activin A-mediated increase in MMP2 secretion (**B**). Untreated cells were used as controls. Each bar represents the mean ± SEM of 3 independent experiments performed in duplicate. Dunnett’s test was used for statistical analysis; * *p* ≤ 0.05; ** *p* < 0.01; *** *p* < 0.001; ^#^ *p* < 0.0001; Act A, activin A; Inh A, inhibin A.

Activin A significantly induced MMP2 secretion in a concentration- and time-dependent manner (Figure 6A). An approximately 2-fold increase was observed after a 24 h stimulation. A stimulation with 5–50 ng/mL of activin A for 48 and 72 h led to an approximately 2- to 5-fold increase in MMP2 secretion (Figure 6A). Conversely, the inhibin A stimulation had no effect on MMP2 secretion (Figure 6B). The simultaneous addition of inhibin A and activin A resulted in a ~20% decrease in activin A-induced MMP2 secretion (Figure 6B). Additionally, activin A stimulation of the 12Z cells resulted in a significant time- and concentration-dependent increase in MMP3 secretion (Figure 6C). An approximately 2-fold increase in MMP3 secretion was observed after 48 h and a ~3- and 4-fold increase after 72 h with 25–50 ng/mL of activin A, respectively (Figure 6C). On the contrary, inhibin A stimulation of the 12Z cells had no effect on MMP3 secretion (data not shown). Taken together, these results imply that activin A promotes the secretion of both MMP2 and MMP3 in endometriotic 12Z cells whereas inhibin A has no effect.

### 3.6. Influence of Recombinant Betaglycan on Cell Viability and Proliferation

**Figure 7:**
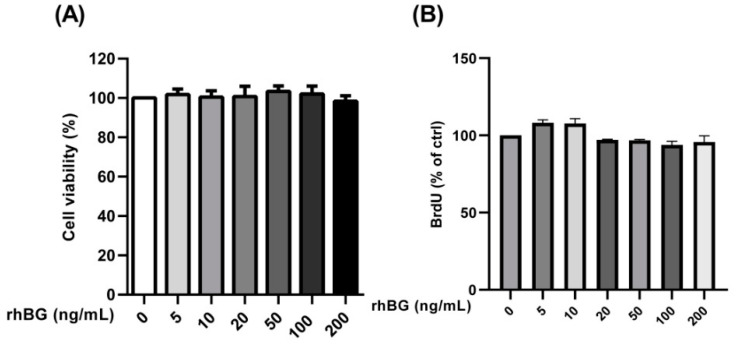
Concentration-dependent effects of recombinant BG (rhBG) on cell viability and proliferation. Endometriotic epithelial 12Z cells were treated with increasing concentrations of the recombinant BG (5–200 ng/mL) and were analyzed for cell viability (**A**) and proliferation (**B**) after 24 h using the CCK-8 and BrdU assay kits, respectively. BG treatment had no effect on the number of viable cells and cell proliferation at the concentrations tested. Untreated cells were used as a control and the mean OD of the control was set to 100%. Each bar represents the mean ± SEM of 3 independent experiments performed in duplicate. Dunnett’s test was used for statistical analysis; rhBG, recombinant betaglycan.

A previous study reported decreased growth rates of murine breast cancer cells following the down-regulation of BG expression [43]. Thus, we analyzed the effects of BG on the viability and proliferation of endometriotic epithelial cells and found no effects at the concentrations tested (Figure 7A,B). Additionally, all concentrations exhibited a viability above 98% indicating that the recombinant BG had no effects on cell viability (Figure 7A).

## 4. Discussion

Recently, we observed that BG is a vital modulator of TGF-β2 signaling in rat Sertoli cells [31] and that TGF-β1/2/3 modulate the shedding of BG in human endometrial cells [30]. In the current study, we demonstrated that activin A suppresses the shedding of BG in human endometriotic cells via the ALK-4/SMAD3 axis but, surprisingly, follistatin did not block activin A activity. Activin A was observed to increase the BG mRNA expression but had no effect on the protein expression. Additionally, inhibin A also reduces BG shedding in endometrial cells. Notably, activin A significantly enhanced the secretion of MMP2 and MMP3 while inhibin A had no effect. Our results also showed that the recombinant BG had no effect on the viability and proliferation of endometriotic cells.

BG serves as a TGF-β accessory receptor or as an antagonist in its soluble form [44,45]. Inhibin binds to activin type II receptors (ACVR2) via its β-subunits and functionally antagonizes activin activity by inhibiting the recruitment of ACVR2; however, to attain high inhibitory potency, inhibin requires the presence of membrane-bound BG [46]. A previous report indicated that BG functions as a tumor suppressor in numerous cell types including endometrial cancer [23] while its role in pathophysiology of endometriosis remains unknown.

### 4.1. Action of Activin A and Inhibin A on BG Shedding in Endometrial Cells

One of the most important roles of BG is to regulate activin signaling through direct interaction with inhibin [13]. Binding of inhibin to BG and ACVR2 results in the formation of a stable complex that sequesters ACVR2 and lessens their accessibility to propagate activin signaling [33]. Recently, we found that TGF-β1/2/3 modulate BG shedding in endometrial epithelial cells [30]. Our present findings demonstrated a significant time- and concentration-dependent reduction in BG shedding with activin A and inhibin A treatment in both endometrial epithelial and stromal cells. Activin A was also observed to enhance the BG mRNA expression but had no effect on the membrane-bound BG protein expression. Although we observed a reduced BG shedding following activin A treatment in endometrial cells, these findings contradict our previous observations using TN4A5 Sertoli cells where activin A treatment significantly induced BG shedding [31]. Accordingly, the functional impact of activin A on BG shedding seems to be both cell-type- and probably context-dependent.

TGF-βs have a greater affinity for BG compared to inhibin although a considerable overlap in the binding sites for TGF-βs and inhibin has been observed within the membrane proximal domain of BG [47,48]. TGF-β was also reported to block access of inhibin A to BG in LβT2 gonadotrope cells, thus directly attenuating the inhibin-mediated antagonism of activin effects [49]. Interestingly, the recombinant inhibin A was reported to antagonize TGF-β2 signaling by causing an endocytic clathrin-independent internalization of the cell surface BG in adrenocortical cells [48].

### 4.2. Mechanisms Involved in Activin A-Mediated Modulation of BG Shedding

Activin binds to two type II serine–threonine kinase receptors, ACVR2A and ACVR2B, resulting in the recruitment and association with two type I activin receptors that are subsequently phosphorylated and activated [15]. The activin type I receptors are activin receptor type-1B (ACVRIB or ALK-4) and activin receptor type-1C (ACVRIC or ALK-7). Results of the present study indicate that the suppression of BG shedding is mediated by ALK-4, generally regarded as the principal activin type I receptor [41], since the ALK-4/5 inhibitor, LY364947, significantly abrogated this effect. Activins are antagonized by follistatin, an activin-binding protein that influences the accessibility of activins to their respective receptors [50]. In this study, the pre-treatment of cells with follistatin had no effect on the activin A-induced regulation of BG shedding at the tested concentration indicating that follistatin is not involved in the activin A-mediated regulation of BG shedding in endometriotic cells. Using Sertoli cells and testicular germ cells, it was demonstrated that the regulation of activin activity by follistatin is cell- and tissue-specific [51]. The authors observed that follistatin inhibited the ability of activin A to stimulate re-aggregation of Sertoli cell monolayers but had no effect on the activin A-mediated incorporation of [3H]-thymidine into Sertoli-testicular germ cell co-cultures.

Our study explored the involvement of the canonical SMAD signaling pathway in the activin A-mediated modulation of BG shedding. Our in vitro SMAD2/SMAD3 gene knockdown experiment demonstrated that the silencing of SMAD3 but not SMAD2 blocked the activin A-mediated suppression of BG shedding. These results were further corroborated using SIS3, a specific inhibitor of SMAD3, which confirmed the involvement of SMAD3 in the activin A-mediated suppression of BG shedding. These results agree with our previous findings using TGF-β1/2 in endometriotic cells in which TGF-β was shown to suppress BG shedding via the ALK-5/SMAD3 pathway [30]. Thus, SMAD3 seems to be the main pathway involved in BG shedding.

Normally, phosphorylated SMAD3 binds to the common SMAD4 prior to the translocation of the complex to the nucleus [15]. The association of the SMAD complex and transcription coactivators with activin-responsive elements (AREs) then leads to the transcription of hundreds of genes [15]. In our case, we speculated about genes encoding matrix metalloproteinases since, using MMP inhibitors, we previously demonstrated that MMPs are involved in inducing the shedding of BG [30,31].

### 4.3. Role of Activin A and Inhibin A in MMP2 and MMP3 Secretion

The over-expression, enhanced secretion, and activity of MMP2 and MMP3 has previously been implicated in pathogenesis of endometriosis [12,35,52,53]. Previously, we demonstrated that TGF-β1 and TGF-β2 promoted the secretion of MMP2, MMP3, and MMP9 in endometrial and endometriotic cells [30,54]. In the present study, we further extended these data, demonstrating that activin A significantly stimulated MMP2 and MMP3 secretion in endometriotic cells. Inhibin A stimulation of the cells had no effect on the secretion of either MMP2 or MMP3, although inhibin was shown to moderately inhibit the activin A-induced secretion of MMP2. A study by Jones et al. [42] also demonstrated an enhanced endometrial secretion of pro-MMP2/3 and active MMP2 with activin A stimulation, whereas inhibin A antagonized these actions in regulating decidualization and trophoblast invasion. Similar observations were made where exogenous activin A enhanced the mRNA expression and gelatinolytic activity of MMP2 in mouse peritoneal macrophages [55]. Additionally, the stimulation of HTR8/SVneo immortalized human extravillous cytotrophoblast (EVT) cells, and primary EVT cells with activin A increased mRNA expression and protein production of MMP2 which was correlated with enhanced trophoblast cell invasion [56].

MMPs are crucial in cyclic changes of the endometrial structure and thickness in the course of the endometrial cycle [12,35,52,53]. Endometrium and peritoneal fluid from women with endometriosis were shown to have elevated levels of MMP-2 and -3 [12,35,52,53]. Endometriosis-induced disruptions in the matrix balance leads to angiogenesis, adhesion formation, ovulatory dysfunction, as well as fertility impairment [12,35,52,53].

### 4.4. Role of Betaglycan in Cell Viability

Studies with both in vitro and in vivo cancer models indicated that BG is a vital modulator of cell growth, migration, angiogenesis, along with apoptosis [22,23]. Thus, we investigated the influence of varying concentrations of the recombinant BG on the viability and proliferation of endometriotic epithelial cells and demonstrated no effect on the number of viable cells as well as on the rate of cell proliferation. Similarly, the restoration of the BG expression in prostate cancer DU145 cells was reported to inhibit the migration and invasion but had no effect on cell proliferation [57]. Moreover, BG was reported to have no effect on cell proliferation or apoptosis of two human granulosa cell tumors (GCT) cell lines, COV434, and KGN [58]. The expression of BG in non-small cell lung cancer (NSCLC) cells expressing normal to low levels of BG showed no effect on cell proliferation [59]. Moreover, in an NSCLC xenograft assay, the over-expression of BG resulted in decreased tumor incidence, tumor growth, as well as tumor invasiveness. In our study, we used both primary and immortalized endometrial cells which are regarded as ideal models to study the molecular and cellular aspects of endometriosis in humans. Future studies are needed to recapitulate our present in vitro findings in an in vivo model.

## 5. Conclusions

**Figure 8:**
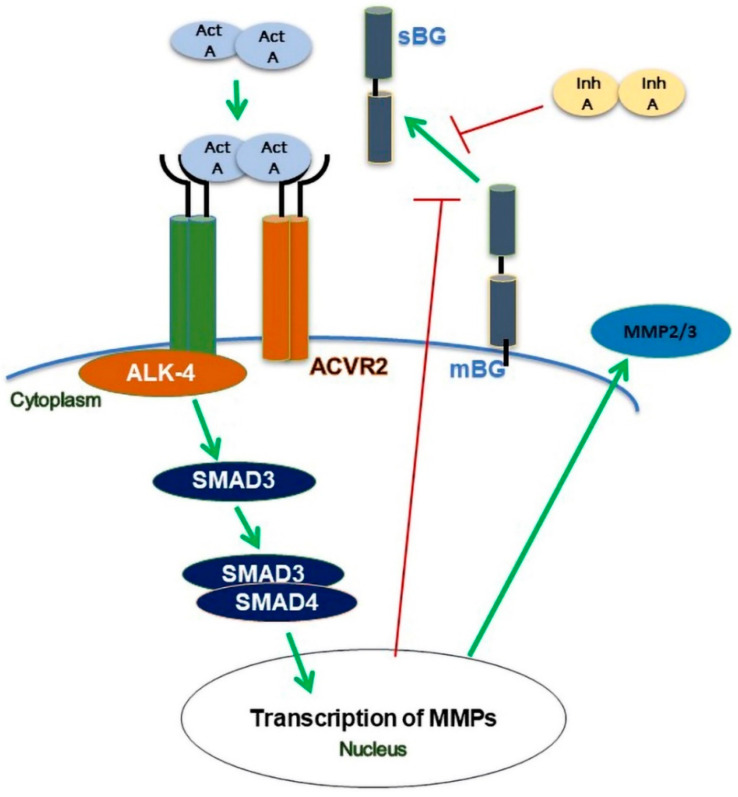
Scheme of modulation of betaglycan (BG) shedding in human endometrial cells. Binding of activin A (act A) to the ALK-4 receptor complex results in a reduction in BG shedding via phosphorylation of SMAD3 but not of SMAD2. Activin A increases the secretion of MMP2 and MMP3. Inhibin A (inh A) decreases the shedding of BG and an activin A-mediated increase in MMP2 secretion.

Collectively, our present in vitro study highlights several novel aspects of activin A/inhibin A and BG in the context of endometriosis. We demonstrated for the first time that activin A suppresses the shedding of BG via the canonical ALK-4/SMAD-dependent pathways (Figure 8). Using epithelial endometriotic cells, we also showed that activin A but not inhibin A enhances the secretion of MMP2 and MMP3. Additionally, we found that the recombinant BG has no effect on the viability and proliferation of epithelial endometriotic cells. These novel findings contribute significantly to further clarify the role of the TGF-β family in the pathophysiology of endometriosis. The strong reduction in BG shedding indicates that the TGF-β ligands require mBG for signaling, since BG is particularly vital in establishing the potency of its ligands on their target cells. Reduced levels of soluble BG may indicate enhanced TGF-β ligand functions, which may in part be linked to the chronic inflammation observed in endometriosis. We propose that BG may represent a potential therapeutic agent and, hence, further studies are required to elucidate its function in endometrial tissue and the peritoneal cavity. The eventual association with endometriosis will undoubtedly provide valuable insights into the development and pathophysiology of endometriosis.

## Data Availability

The raw data are available from the corresponding author upon request.
